# Recovery of benthic macroinfauna six years after dredging

**DOI:** 10.1371/journal.pone.0332089

**Published:** 2025-09-17

**Authors:** David Samuel Johnson, Manisha Pant, Andrew Nemeth, Emelie Foster, Adam Gartelman, Stacy Calhoun-Grosch, Kehui Xu, Brian J. Roberts, James Nelson

**Affiliations:** 1 Virginia Institute of Marine Science, William & Mary, Gloucester Point, Virginia, United States of America; 2 Department of Marine Sciences, University of Georgia, Athens, Georgia, United States of America; 3 Coastal Studies Institute, Louisiana State University, Baton Rouge, Louisiana, United States of America; 4 Department of Oceanography and Coastal Sciences, Louisiana State University, Baton Rouge, Louisiana, United States of America; 5 Louisiana Universities Marine Consortium, Chauvin, Louisiana, United Stataes of America; University of Basrah, IRAQ

## Abstract

In soft-sediment communities, benthic macroinfauna can recover quickly (sometimes < 1 year) from dredging. However, it’s unclear how recovery is influenced if dredging dramatically changes abiotic factors. We determined recovery of benthic macroinfauna from dredging on a sandy shoal (Ship Shoal, Louisiana, USA), six years after dredging. Because the dredged area infilled with mud instead of sand and changed local abiotic conditions, we hypothesized that the benthic community had not recovered. Our results support this hypothesis. Although the dredged community had, on average, 160% greater density and 170% greater macroinfaunal biomass than the reference community, these increases were driven by the opportunistic and seasonally variable dwarf clam, *Mulinia lateralis*. Community structure differed starkly between the regions. Haustoriid amphipods and amphioxus (*Branchiostoma floridae*) dominated the reference community. However, these taxa were largely absent from the dredged community, which was dominated by opportunistic species such as *M. lateralis* and the fringe-gilled mudworm *Paraprionospio pinnata*. Community differences were largely driven by bottom-water dissolved oxygen and sediment grain size. Our results suggest that the dredged areas are still in a transitional phase of recovery after six years – dominated by opportunistic, hypoxia-tolerant species. Our results suggest that abiotic factors such as grain size and hypoxia are important in structuring benthic communities and their recovery rates. Thus, to achieve faster recovery of the biological community, dredging should be conducted in a way that minimizes long-term impacts on abiotic conditions.

## Introduction

A common practice worldwide to restore coastal habitats such as barrier islands, beaches, and wetlands is to use sediment dredged offshore. In the U.S., shallow-water (< 30 m), sandy shoals are a rich source of sediment for coastal restoration projects [1]. Although building up shorelines benefits coastal habitats, the dredging itself impacts the shoal’s biological community. The question is, how long does this impact last? Macroinfauna (i.e., sediment-dwelling animals such as worms >500 µm) are key to evaluating the recovery of dredging impacts because dredging results in complete removal of animals and during recovery they are sensitive to changes in sediment grain size, dissolved oxygen, and organic matter inputs that occur with dredging [2,3], [4,5]. In addition, because macroinfauna are critical links in food webs that transfer energy from primary producers to predators [6,7], changes in the benthic macroinfaunal community due to dredging may indirectly influence the biomass and community structure of predators such as fishes, shrimps and crabs [8].

Located off the coast of Louisiana, Ship Shoal is a substantial source of sediment for restoration projects in Louisiana. Recently, the eastern end of Ship Shoal has been dredged for sediment used in coastal restoration projects in Louisiana. We refer to this borrow area as the Caminada dredge pit. It was dredged from 2014–2016 (see Methods for details). Since then, its sediment grain size and bottom-water dissolved oxygen has decreased, while sediment organic matter has increased [9–11]. For instance, as suspended mud from the Mississippi and Atchafalaya Rivers passes over Ship Shoal, it falls into the pit and has shifted its sediments to finer sediments [12,13]. These changes in sediment and water chemistry may prevent quick recovery of the macroinfauna community (relative to undredged areas).

In their survey of Ship Shoal’s benthic macroinfauna in 2006, prior to any dredging, Dubois et al. [2] found its infaunal diversity, abundance, and biomass exceptionally rich. Its community was dominated by polychaetes, crustaceans (e.g., amphipods), and amphioxus (also known as lancelets, subphylum Cephalochordata, *Branchiostoma floridae*) [2]. Dredging impacts were not immediately assessed on the benthic macroinfauna in the Caminada dredge pit, but we can assume the infauna experienced similar immediate impacts seen from other dredging studies: up to a 90% loss of abundance and biomass, diminished biodiversity, and a dramatic shift in community structure, including species identity [14,15].

Benthic infaunal communities in sandy sediments typically can recover <1 year to 3 years after dredging (as reviewed in [14,15]). Because Ship Shoal is sandy, we could expect quick recovery of the benthic infauna in the Caminada dredge pit. However, because of the recent change in surficial sediment type and water chemistry, which can influence infaunal communities [3,11], the Caminada infaunal community has likely not fully recovered compared to undredged areas. For instance, in their study of a dredge pit between Calcasieu and Sabine Passes offshore from coastal Louisiana (*ca.* 200 km northwest of Ship Shoal), Palmer et al. [16] found that the benthic infaunal community had not recovered after three years, and this lack of recovery was associated with the accumulation of finer sediments.

The Pearson-Rosenberg model of responses of benthic infauna to disturbances is useful for understanding its recovery after dredging. Originally, the Pearson-Rosenberg model was developed based on macrobenthic responses to organic (e.g., sewage) enrichment of sediments [17]. However, it can be generalized to a variety of disturbances including dredging [18]. In this way, the model states that benthic communities move through three progressive community phases after a disturbance: 1) A colonization phase with high abundances of small-bodied, opportunistic species, 2) a transitional community where species diversity increases, and 3) an equilibrium (aka ‘climax’) community where the dominant species change from disturbance-tolerant to disturbance-sensitive species.

The primary objective of our study is to determine if the benthic macroinfaunal community has recovered since dredging in the Caminada pit concluded in 2016. To assess recovery, we compared macroinfaunal communities in dredged areas to nearby undredged areas (referred to as reference areas) five-six years after dredging. We hypothesized that because the dredge pit infilled with finer sediments and has different chemical properties, the benthic community in the dredged areas will not have recovered. For recovery, we considered the following criteria: the dredged macroinfaunal community must have at least 80% of the 1) total abundance and 2) community biomass of the reference community [18] and 3) must also resemble the reference community structure (i.e., similar species and relative abundance).

## Methods

### Study area

Ship Shoal is a reworked deltaic headland of the wandering Mississippi River delta plain [19], located approximately 15 km off the Isles Dernieres, Louisiana, U.S.. Based on historical records, Ship Shoal was Ship Island, a former barrier island, that was submerged between 1827 and 1853 [12]. Ship Shoal is approximately 50 km long and 2–10 km wide [20] with water depths ranging from 4–10 m, which is shallower than the surrounding 30 + m depths of the Louisiana shelf [19]. Ship Shoal is a considerable offshore sand resource, containing 1.22 billion m^3^ of fine sand [10].

Recently, the eastern end of Ship Shoal – the focus of this study – has been dredged for several restoration projects. The Caminada borrow area was dredged for the Caminada Headland Beach and Dune Restoration Project in two sections (aka increments) of the pit. Increment I, the western side of the pit, was dredged from June 2013 – January 2015 and Increment II, the eastern side, was dredged from May 2015 – November 2016 to a depth 7–14 m from the seabed. For this study, we designated two regions, the Caminada dredge pit and a reference region. Our sampling spanned both increments of the Caminada dredge pit. Dubios et al. [2] found that macroinfauna abundance, biomass, and community composition were similar among samples taken on the eastern side of Ship Shoal, which is where our reference and dredge pit regions are. Thus, our designated reference and dredged areas had similar fauna prior to dredging.

### Benthic infauna collections

Macrofauna were collected using a GOMEX box corer (30 cm x 30 cm) aboard the *R/V Acadiana* that ports at the Louisiana University Marine Consortium (LUMCON) in Chauvin, Louisiana, U.S. In each region (reference, Caminada), 3 box cores were taken within each of three 500 m x 500 m sites (n = 9/Region/Sampling trip). We sampled in the spring (March/April), summer (June/July), and fall (October/November) of 2021 and 2022. On board, cores were immediately fixed in 5% buffered formalin. After no more than two days, samples were sieved on 500 μm sieve to retain macroinfauna. After 7 days, macrofauna were transferred to 70% ethanol in the laboratory and identified to lowest possible taxon. Although recorded, juvenile fish (n = 2), insects (n = 4) and spiders (n = 2) were removed prior to analysis because they are inadequately sampled (fish) or terrestrial contaminants (insects and spiders).

We estimated the biomass of benthic infauna based on ash-free dry weights (AFDW). Animals were grouped as bivalves, polychaetes, amphipods, gastropods, and amphioxus (cephalochordates) per sample and dried at 40°C until a constant weight (typically 48 hours). Then samples were burned in a muffle furnace at 500 °C for 4 hours. Bivalves included the hyperabundant *M. lateralis* and rarely found bivalve species. *M. lateralis* was 92% of the total bivalve biomass. Decapods such as hermit crabs and blue crabs were excluded because although rare, when present a single decapod’s biomass exceeded the remaining community biomass.

### Environmental variables

At the same time benthic infauna were sampled, we sampled environmental characteristics. These variables were used to determine if they are driving the infaunal community structure. We do not present regional comparisons of these variables here because they can be found elsewhere (e.g., sediment: [12], water chemistry and sediment organic matter: [21]). Based on known factors that influence infaunal community structure (e.g., [2,22]), we selected the following variables: bottom-water dissolved oxygen, benthic algal biomass (based on chlorophyll *a* concentration), percent organic matter of sediment, median grain size, and percent sand. See S1 File for methods on these collections.

### Statistical analyses

#### Univariate analyses.

To determine the effect of dredging on benthic infauna populations, we focused on the abundances of the four most common groups: the dwarf surf clam, *Mulinia lateralis*, the polychaete *Paraprionospio pinnata*, the cephalochordate *Branchiostoma floridae*, and Haustoriidae amphipods. Together, these animals made up 80% total community abundance across both regions. We also compared the total abundance of infauna communities with and without *M. lateralis*. This clam was the numerically dominant species across all samples (43% of the global community); however, it peaked seasonally and was only abundant in the Caminada dredge pit. For each of the above, we fit generalized linear mixed models (GLMMs) in R version 4.4.2 (R Core Team 2024) using the glmmTMB package version 1.1.10 [23]. In these models, the abundance was the response variable, region (reference and Caminada) and Sampling Period (sampling trips) were fixed effects, and sample site within Region was a random effect. We included an interaction term between region and period, but such interaction models did not fit for some taxa. We fitted models assuming Poisson and two types of negative binomial distributions, and the preferred models were chosen based on AICc and model diagnostic plots. Here, model fit was evaluated based on examination of the residuals using the DHARMa package version 0.4.7 [24] in R.

#### Multivariate analyses.

We conducted permutational multivariate ANOVAs (PERMANOVAs) to determine if there is a statistical difference between reference and Caminada macroinfaunal communities. A PERMANOVA is a non-parametric, multivariate method that does not require all variables to meet the assumption of normality that is required for MANOVAs [25]. The variables in this case are the abundance of infaunal species. To test for the effect of time and dredging on the benthic infauna, we first conducted a 2-way PERMANOVA with Sampling Period and Region (reference, Caminada) as fixed effects. Because there was a significant interaction between Sampling Period and Region, we followed this with a series of 1-way PERMANOVAs for each sampling trip with Region as the predictor. Prior to PERMANOVA analysis, data were fourth-root or Hellinger-transformed to down-weight large values. Bray-Curtis index of similarity was used to develop each resemblance matrix. PERMANOVA models were fitted using the R package vegan version 2.6–8 [26].

To visualize differences in communities between the two regions, we developed non-metric multi-dimensional (nMDS) ordination plots for each sampling trip using the R package vegan version 2.6–8 [26]. nMDS is a permutational ordination technique where samples are represented as points in a plot, such that points that are closer together in the plot have more similar assemblages than those farther apart. The data distortion due to the reduction of the multivariate assemblage data into few (typically 2 or 3) dimensions is often represented by the metric “stress.” Lower stress values are better, and stress values <0.20 are considered acceptable.

To determine which, if any, environmental variables are driving macroinfaunal community structure, we conducted a redundancy analysis (RDA) in R package vegan version 2.6–8 [26]. RDA is an extension of multiple regression that models the explanatory power of environmental variables on species assemblages [27]. We chose the following environmental variables that are known to influence infaunal community structures: bottom-water dissolved oxygen, benthic algal biomass (based on chlorophyll *a* concentration), percent organic matter of sediment, median grain size, and percent sand. Because not all the environmental data came from the same box cores as the infauna box cores (though near each other), we took the average of the triplicate box cores taken at each site within a Region (Caminada and reference). Thus, for these analyses, n = 3/Region/Sampling Period. Median grain size and percent sand were collinear in the RDA (variance inflation factor > 10); therefore, median grain size was removed as a predictor from the RDA. Two separate RDA analyses were conducted. The first conditioned on Region and Sampling Period (i.e., controlled for the effects of Region and Sampling Period), and then tested for the effect of the environmental variables. If this test is significant, we concluded that environmental variables explain additional variation not explained by Region and Sampling Period alone. The second RDA was conditioned on environmental variables and then tested for the effect of Region and Sampling Period. If this test is significant, we conclude that differences between community composition in Caminada and reference sites are not due alone to their environmental differences. For each, we rank-transformed the environmental variables bottom-water dissolved oxygen, percent organic matter, and percent sand to account for drastic difference in their variances between Caminada and reference.

## Results

Across all samples, we identified 100 taxa from 20,245 individuals collected from our sampling of the eastern side of the shoal.

### Population analyses

**Fig 1:**
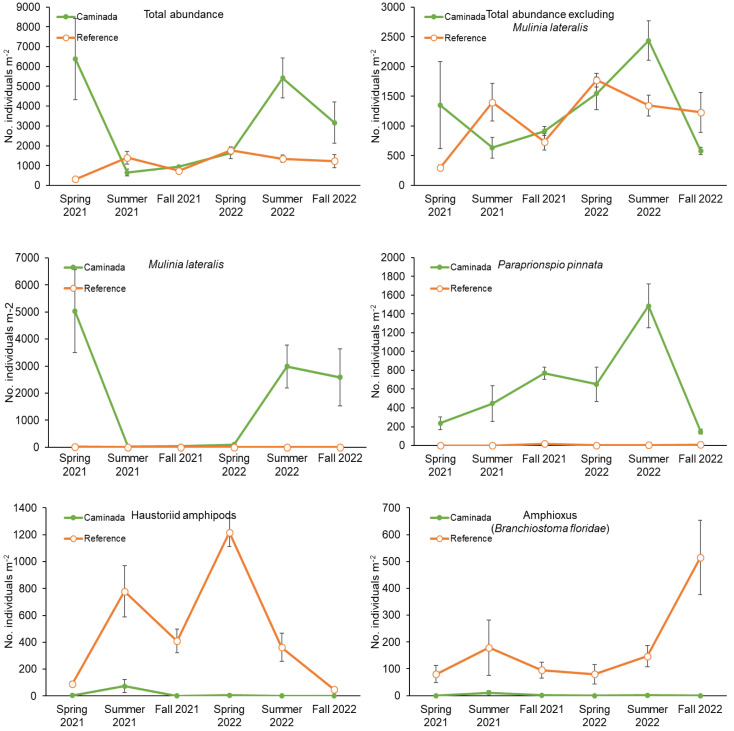
Mean (±1 SE, n = 9) density of dominant macroinfauna in the Caminada dredge pit and the reference region on Ship Shoal.

When pooled across all sampling periods, Caminada had, on average, 160% more total infauna than the reference community, which exceeds the 80% criteria for recovery. The magnitude of the increase of infauna depended on what season the samples were taken (GLMM negative binomial distribution model, Sampling Period x Region interaction, χ^2 ^= 113.5, df = 5, p = 2.2 x 10^−16^). The mean total abundance of benthic macroinfauna was 1900% higher in Caminada than in reference in Spring 2021 (Fig 1). This was due to the extraordinary densities of the dwarf surf clam, *Mulinia lateralis*, which represented 79% of the Caminada community by abundance in Spring 2021 (mean density = 5021 clams m^-2^, Fig 1). Total abundance, on average, remained similar across regions until Summer 2022 when Caminada had densities 300% greater than reference abundances. For Caminada, this was again due to the presence of *M. lateralis*. When total abundance was analyzed in the absence of *M. lateralis*, Caminada still had 10% more infauna than the reference community.

The density of *M. lateralis* was 45,000% higher in Caminada than in reference when pooling across all sampling periods (Caminada: global mean = 1790 clams m^-2^; reference: global mean = 4 clams m^-2^, GLMM negative binomial distribution model, Region χ^2 ^= 257.0, df = 1, p = 2.2 x 10^-16^). *Mulinia lateralis* was rarely found in reference (range = 0–44 clams m^-2^) and hyper-abundant, although seasonally variable, in Caminada (range = 0–13,210 clams m^-2^, Sampling Period χ^2 ^= 15.9, df = 5, p = 2.2 x 10^-16^). *Mulinia lateralis* densities peaked in Caminada in Spring 2021, crashed in Summer 2021 and peaked again in Summer 2022 (Fig 1).

Pooling across all sampling periods, *Paraprionospio pinnata* densities were 10,300% greater, on average, in Caminada than in reference (Caminada: global mean = 623 worms m^-2^; reference: global mean = 6 worms m^-2^). The magnitude of the increase of infauna depended on when samples were taken. It was the dominant polychaete in Caminada with a peak mean density of 1485 worms m^-2^ in Summer 2022 (Sampling Period x Region interaction: GLMM negative binomial distribution model, χ^2 ^= 15.9, df = 5, p = 0.007, Fig 1). Its population crashed in all regions in Fall 2022 (Fig 1). It was rarely found in the reference region throughout the study (range = 0–99 worms m^-2^).

In contrast, amphioxus (*Branchiostoma floridae*) densities were, on average, 99% lower in Caminada than reference when pooled across all sampling periods, (Caminada: global mean = 2 individuals m^-2^; reference: global mean = 183 individuals m^-2^, GLMM negative binomial model, Region, χ^2 ^= 65.4, df = 1, 5.7 x 10^−16^). It was most abundant in the reference region peaking with a mean density of 512 individuals m^-2^ in Fall 2022 (Fig 1, GLMM negative binomial model, Sampling Period, χ^2 ^= 41.3, df = 5, p = 8.0 x 10^−8^). It was virtually absent in Caminada (range = 0–44 individuals m^-2^).

Haustoriid amphipods were most abundant in the reference region throughout most of the study but crashed in Fall 2022 (GLMM negative binomial model Sampling Period x Region interaction, χ^2 ^= 15.4, df = 5, p = 0.009). They peaked in Spring 2022 with a mean of 1217 amphipods m^-2^ (Fig 1). Haustoriid amphipods were virtually absent from Caminada, with them occurring only in Summer 2021. As a result, amphipod densities were 97% lower in Caminada than in reference.

### Community analyses

**Fig 2:**
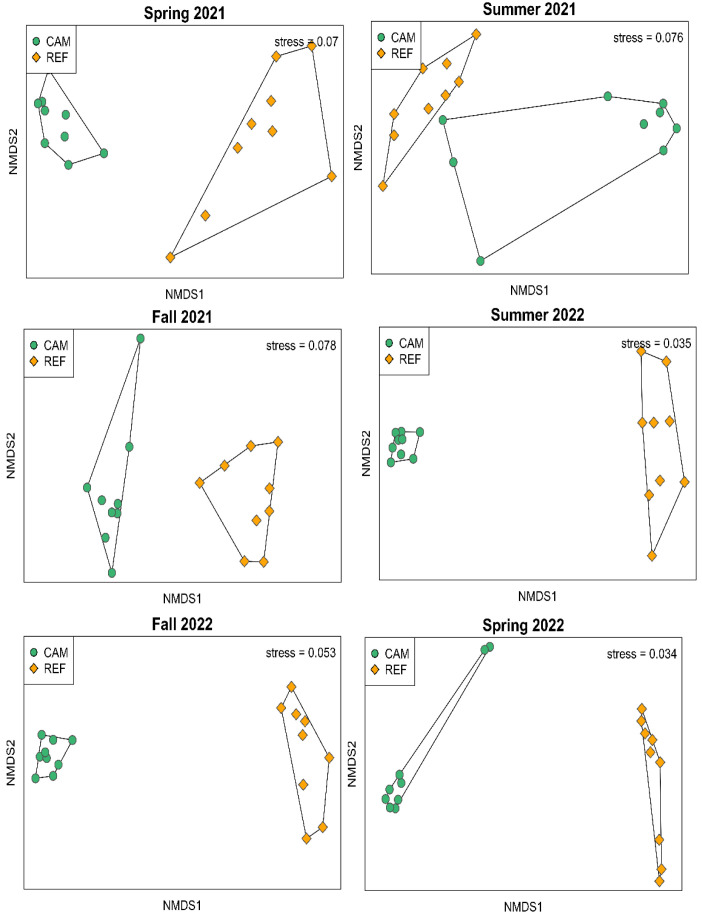
Non-metric multidimensional scaling plots show that benthic macroinfauna communities in the Caminada dredge pit (CAM) and the reference region (REF) were dissimilar from Spring 2021 to Fall 2022.

Based on the top 5 numerically dominant species across all sampling periods, the reference community was characterized by haustoriid amphipods, amphioxus (*Branchiostoma floridae*), the polychaetes *Travisia hobsonae* and *Spiophanes bombyx*, and the phoronid *Phoronis architecta* (Table 1). The Caminada community was characterized by the bivalve *Mulinia lateralis*, the polychaetes, *Paraprionospio pinnata*, *Ampharete* sp., *Cossura longocirrata*, and *Sigambra tentaculate* (Table 1). Based on nMDS plots and PERMANOVA analysis, the reference and Caminada communities were distinctly different from each other based on composition (who is present) and relative abundance. This difference persisted throughout the study (Fig 2, PERMANOVA for Region effect (reference vs. Caminada), p < 0.01 for all Sampling Periods). Thus, the macroinfaunal community in Caminada fails to meet the criteria for recovery community analysis.

**Table 1:** Relative abundance (% of total abundance) of top 5 numerically-dominant species by region on Ship Shoal. Relative abundance based on all samples pooled across all sampling periods. See S1 Table for full list.

Reference			Caminada		
Classification	Taxon	(%)	Classification	Taxon	(%)
Amphipod	*Haustoriidae* amphipods	42.6	Bivalve	*Mulinia lateralis*	59.1
Cephalochordate	*Branchiostoma floridae*	16.1	Polychaete	*Paraprionospio pinnata*	20.5
Polychaete	*Travisia hobsonae*	9.8	Polychaete	*Ampharete sp*	3.7
Phoronid	*Phoronis architecta*	2.1	Polychaete	*Cossura longocirrata*	2.4
Polychaete	*Spiophanes bombyx*	1.8	Polychaete	*Sigambra tentaculata*	2.2

**Fig 3:**
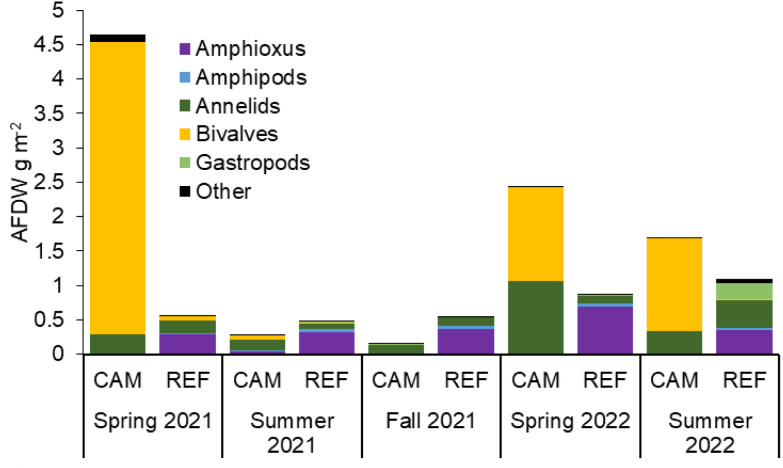
Mean ash-free dry weight (AFDW) of benthic infauna groups from Ship Shoal. CAM = Caminada dredge pit, REF = reference region.

When averaging across seasons and years, the Caminada infaunal community had 170% more biomass than the reference community when bivalves were present (Fig 3). This increase was driven by the presence of *M. lateralis*, which accounts for ~80% of the total community biomass in Caminada (averaged across all seasons and years). When *M. lateralis* was abundant in the Caminada community (i.e., Spring 2021, 2022, Summer 2022), it constituted 55–91% of the community biomass and the Caminada community biomass was 96–736% higher than the reference community biomass. When bivalves are largely absent (i.e., Summer and Fall 2021), infaunal community biomass in Caminada was 40–75% less than in the reference region.

**Fig 4:**
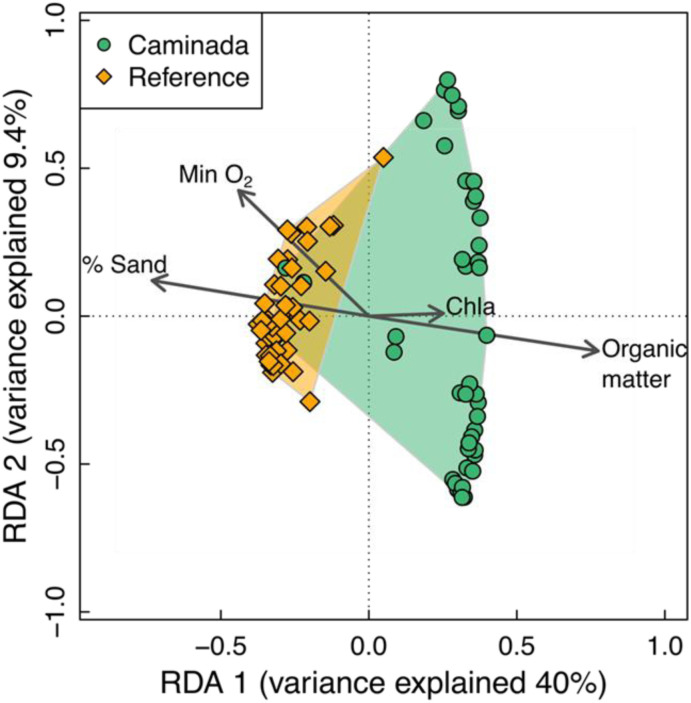
An ordination plot showing results of a redundancy analysis (RDA) conducted on benthic macroinfauna communities in reference (orange diamonds) and Caminada sites (green dots) on Ship Shoal. Minimum bottom-water dissolved oxygen (Min O_2_), percent sand (% Sand), benthic chlorophyll a (Chla) and mean organic matter in the sediment (Organic matter) significantly explain the variation between the reference and Caminada macroinfaunal communities. As minimum bottom dissolved oxygen and percent sand increased, the macroinfaunal community favored a reference community. As chlorophyll a content and organic matter increased, the infaunal community favored a Caminada community.

The environmental variables and region explained 59.5% of the total variation in the macroinfaunal community composition across sites. Overall, the RDA analysis showed that both Region and environment factors independently affected benthic infaunal community structure in Ship Shoal. RDA conditioned on environmental variables was statistically significant (F = 7.8, df = 5,80, p = 0.001), suggesting that environment alone does not explain the differences in community structure between Caminada and reference. RDA conditioned on region and sampling period was also statistically significant (F = 3.5, df = 4,80, p = 0.001), suggesting that environmental variables had a unique effect, in addition to the effect of Region. Benthic chlorophyll *a* (RDA, p = 0.027), percent organic matter (p = 0.002), percent sand (RDA, p = 0.016) and minimum bottom water dissolved oxygen (RDA, p = 0.002) all significantly explained the variation between the reference and Caminada macroinfaunal communities (Fig 4).

## Discussion

Our study concluded six years after the Caminada dredge pit was last dredged. We assessed macroinfauna recovery by comparing areas in the Caminada dredge pit to undredged areas based on the criteria of abundance, biomass, and community structure. We hypothesized that the dredged areas had not recovered because of the infilling of mud and changed water properties. Our results suggest that the Caminada dredge pit has not recovered because the communities are starkly different. In terms of total abundance and biomass of animals, the Caminada community matched or exceeded the reference community. The communities were distinctly different from each other throughout the study, with different species dominating each region. Amphipods, amphioxus (*Branchiostoma floridae*), and polychaetes (*Travisia hobsonae*, *Spiophanes bombyx*) dominated the reference community in terms of abundance. These taxa were largely absent from the Caminada community, which were numerically dominated by polychaetes such as the fringe-gilled mudworm *Paraprionospio pinnata* and seasonal bursts of the bivalve *Mulinia lateralis*. Our results suggest that the differences in communities are driven by bottom-water dissolved oxygen levels, sediment grain size (i.e., % sand), sediment organic matter and benthic algal content. The Caminada pit favors a clam-polychaete community and the reference region favors an amphipod-amphioxus-polychaete community.

The reference community was numerically-dominated by amphipods (particularly family Haustoriidae) and amphioxus. In their shoal-wide survey, prior to any dredging, Dubois et al. [2] found amphipods and amphioxus abundant and frequently throughout Ship Shoal, including the eastern shoal where the reference areas and the Caminada dredge pit are. Although amphipods dominated the abundance in the reference community, the commuminty biomass was dominated by amphioxus. Amphipods in the Haustoriidae family, like those found on Ship Shoal, are often referred to as ‘sand-burrowing’ amphipods [28] and are typical of sandy beaches [29]. Given that the reference region is sandier than the Caminada dredge pit [12, 10] it is not surprising that amphipods dominate this area. Amphioxus, which is the cephalochordate *Branchistoma floridae*, is also a sand-dwelling species. While it can swim, it swims short distances and buries its tail into the sand with its head at the surface to filter feed where it eats microplankton and organic matter [30].

*Mulinia lateralis* and *P. pinnata* are opportunistic species whose abundances commonly spike after sediment-scouring disturbance events like dredging and hurricanes [31–33]. In the Caminada community, the fringe-gilled mudworm *P. pinnata* dominated, with densities exceeding those of the reference community. It is a suspension and surface deposit feeder that feeds on algae and detritus [34,35]. As indicated by its common name, “fringe-gilled *mudworm*”, this polychaete is most abundant in mud and muddy sand [35] and tolerates hypoxia well [36].

During the spring and summer seasons, the dwarf surf clam *M. lateralis* densities exploded in Caminada but not in reference. Like abundance, the community biomass in Caminada was dominated by *M. lateralis* when present. *Mulinia lateralis* is an opportunistic bivalve found in silt-clay sediments that are often unstable [37,38]. It has high fecundity and short generation times that can generate dramatic temporal fluctuations [37,39,40]. *M. lateralis* populations are notoriously episodic, booming and crashing for years as a result of changes in predation, food availability, recruitment, disease, temperature, dissolved oxygen, and salinity [40,41,42,43]. Mid-to-late summertime hypoxia, which is prevalent in the northern Gulf of Mexico, may explain the loss of *M. lateralis* in the fall. *Mulinia lateralis* can be short-lived in hypoxia/anoxia [42]. At the same time, shoals are important foraging grounds for nekton. We suggest that that this bivalve’s window of opportunity in Caminada is in the late spring to early summer when it can take advantage of spring phytoplankton blooms and it crashes in the mid-to-late summer due to prolonged seasonal hypoxia and predation by fishes and shrimps.

### Community differences

The Caminada dredge pit has not recovered in terms of the macroinfaunal community, which is starkly different than the reference community. Based on our redundancy analysis, bottom-water dissolved oxygen, sediment grain size (% sand), and sediment organic matter are key drivers of this difference. Dubois et al. [2] found that grain size and dissolved oxygen best explained the variation in community structure and assemblages on Ship Shoal prior to dredging. Currently, the reference region is > 99% sand (<1% mud) and the Caminada pit is 36–56% sand (44–64% mud) [12]. As a result, the finer, lower-oxygen, more organic-rich sediments of the Caminada dredge pit favor opportunistic clams and polychaetes and the sandier, well-oxygenated, organic-poorer sediments of the reference region favor larger species such as amphipods, amphioxus, and polychaetes.

Dredging areas in low energy-environments can lead to areas with changed physical and chemical properties [44,45], leading to a shift in the benthic community [16,46]. Like our study, infaunal communities in coastal France shifted from one dominated by amphioxus (*B. laceolatum*) to one dominated by polychaetes after dredging [46]. In a study of dredging *ca.* 200 km northwest of Ship Shoal, Palmer et al. [16] found that the dramatic infaunal community shift was associated with the accumulation of finer silts and clays. Ship Shoal, in particular, is susceptible to infilling by finer sediment because of silts and clays delivered by the Mississippi and Atchafalaya Rivers.

### Food-web implications

Benthic macroinfauna are vital prey for predators that use Ship Shoal including hardhead catfish (*Ariopsis felis*), blue crabs (*Callinectes sapidus*), brown *(Farfantepenaeus aztecus)* and white *(Litopenaeus setiferus)* shrimp, red (*Sciaenops ocellatus*) and black drum (*Pogonias cromis*), snappers (*Lutjanus* spp.), and cownose rays (*Rhinoptera bonasus*) [47–49]. These predators, in turn, become prey for larger predators such as blacktip sharks (*Carcharhinus limbatus*). Many of these predators are opportunistic but it’s unclear whether predators are taking advantage of *M. lateralis* as prey in the Caminada dredge pit.

The more variable prey biomass in the Caminada dredge pit is likely influencing food-web dynamics. For instance, the benthic-feeding hardhead catfish, *Ariopsis felis*, has a larger trophic niche in the Caminada dredge pit versus the reference region suggesting that these predators are using a greater variety of benthic prey in the dredged areas [48]. Diet expansion is a common strategy of generalist predators to deal with prey variability [50].

## Conclusions

Our results suggest that the benthic macroinfauna in the Caminada dredge pit has not recovered six years after dredging. Although the average total abundance and community biomass of the Caminada community matches or exceeds the reference community, these two communities are vastly different in composition and structure. These differences are likely driven by the accumulation of mud and organic matter in the Caminada pit. The deepening of the pit area (originally ~6 meters deeper that has filled in so it is now ~ 4 meters deeper than the reference region) allows it to infill with finer sediments and more organic material. It does not mix as well by currents as the reference region which likely leads to lower dissolved oxygen concentrations including hypoxia. Based on the Pearson-Rosenberg model, the dredged areas appear to be in a transition phase, dominated by opportunistic species. How long will these communities differ? Likely until the surface sediments in the dredge pit, benthic oxygen levels and sediment organic matter are equivalent to the rest of the shoal (e.g., oxygen-rich, > 99% sand). Based on rates of infilling alone it will take at least another 50 years [12]. To allow for quicker recovery of benthic infauna for future dredging in low-energy areas susceptible to infilling of finer grain sizes and organic matter, we recommend dredging practices that prevent that infilling such as dredging at shallower depths.

## Supporting information

S1 File Methods for environmental variables. (DOCX)

S1 Table Relative abundance (% of total abundance) of benthic macroinfauna by region on Ship Shoal. Relative abundance based on all samples pooled across all sampling periods.
(DOCX)
