# Supplementary material for: Recovery of benthic macroinfauna six years after dredging
Source: PLoS One. 2025 Sep 17;20(9):e0332089. doi: 10.1371/journal.pone.0332089 (PMC12443283; doi:10.1371/journal.pone.0332089)
Supplement: S1 File — (DOCX) [file pone.0332089.s001.docx]

**Supplementary Information** **File 1:** Methods for environmental variables

Cores for sediment analysis (grain size and percent sand) for the top 1-cm were extracted from benthic infauna box cores with a 60 mL cut-off syringe. From box cores taken adjacent to benthic infauna cores, surface (1 cm) sediment samples were collected with a 60 mL cut-off syringe from each core to determine sediment bulk density, water content, and organic matter content (via loss on ignition method) following protocols described in Marton and Roberts (2014). Surface (0.5 cm) sediment samples were collected with a 12 mL cut-off syringe for determination of benthic chlorophyll and phaeopigment concentrations. Benthic chlorophyll was extracted in 90% acetone solution for 24-48 hours prior to analysis on a Turner Designs 10-AU fluorometer using procedures described in Roberts and Doty (2015). Bottom-water dissolved oxygen was determined from CTD casts with an SBE 19plus V2 SeaCAT profiler CTD performed at the site of box cores.
