# Supplementary material for: Recovery of benthic macroinfauna six years after dredging
Source: PLoS One. 2025 Sep 17;20(9):e0332089. doi: 10.1371/journal.pone.0332089 (PMC12443283; doi:10.1371/journal.pone.0332089)
Supplement: S1 Table — Relative abundance based on all samples pooled across all sampling periods. (DOCX) [file pone.0332089.s002.docx]

**Supplementary Table 1**: Relative abundance (% of total abundance) of benthic macroinfauna by region on Ship Shoal. Relative abundance based on all samples pooled across all sampling periods.

| Reference | | |  | Caminada | | |
| --- | --- | --- | --- | --- | --- | --- |
| Classification | Taxon | (%) |  | Classification | Taxon | (%) |
| Amphipod | *Haustoriidae* amphipods | 42.6 |  | Bivalve | *Mulinia lateralis* | 59.1 |
| Cephalochordate | *Branchiostoma floridae*  (aka amphioxus) | 16.1 |  | Polychaete | *Paraprionospio pinnata* | 20.5 |
| Polychaete | *Travisia hobsonae* | 9.8 |  | Polychaete | *Ampharete sp* | 3.7 |
| Phoronid | *Phoronis architecta* | 2.1 |  | Polychaete | *Cossura longocirrata* | 2.4 |
| Polychaete | *Spiophanes bombyx* | 1.8 |  | Polychaete | *Sigambra tentaculata* | 2.2 |
| Polychaete | *Mediomastus californiensis* | 1.7 |  | Polychaete | *Magelona* sp H | 2.2 |
| Crustacean | *Oxyurostylis smithi* | 1.7 |  | Polychete | *Ampharete oculata* | 1.2 |
| Polychaete | *Nereididae* | 1.7 |  | Phoronid | *Phoronis architecta* | 1.2 |
| Echinoderm | *Amphipholis squamata* | 1.3 |  | Polychaete | *Gyptis vittata* | 0.9 |
| Polychaete | *Scoloplos rubra* | 1.3 |  | Polychaete | *Paramphinome* sp B | 0.8 |
| Cnidaria | *Sea anemone* | 1.1 |  | Crustacean | *Oxyurostylis smithi* | 0.6 |
| Polychaete | *Magelona* sp. H | 1.1 |  | Amphipod | *Haustoriidae* | 0.4 |
| Polychaete | *Sthenelais limicola* | 1.0 |  | Polychaete | *Diopatra cuprea* | 0.4 |
| Amphipod | *Amphipod A* | 1.0 |  | Polychaete | *Caulleriella sp* | 0.3 |
| Amphipod | *Ampelisca sp* | 0.9 |  | Polychaete | *Phyllodoce mucosa* | 0.3 |
| Polychaete | *Cirrophorus lyra* | 0.9 |  | Bivalve | Pandoridae | 0.3 |
| Polychaete | *Onuphis eremita oculata* | 0.8 |  | Bivalve | *Macoma sp* | 0.3 |
| Polychaete | *Diopatra cuprea* | 0.7 |  | Crustacean | Shrimp | 0.2 |
| Polychaete | *Dispio uncinata* | 0.7 |  | Polychaete | Nereididae | 0.2 |
| Polychaete | *Capitellidae* | 0.6 |  | Polychaete | *Scoloplos sp* | 0.2 |
| Polychaete | *Phyllodoce mucosa* | 0.5 |  | Polychaete | *Spiophanes bombyx* | 0.2 |
| Polychaete | *Aricidea fragilis* | 0.5 |  | Bivalve | *Solen viridis* | 0.1 |
| Polychaete | *Apoprionospio dayi* | 0.5 |  | Crustacean | Copepod | 0.1 |
| Polychaete | *Caulleriella sp* | 0.5 |  | Amphipod | *Amphipod A* | 0.1 |
| Nematoda | Nematode | 0.5 |  | Polychaete | *Onuphis eremita oculata* | 0.1 |
| Polychaete | *Mesochaetopterus capensis* | 0.5 |  | Polychaete | *Apoprionospio sp* | 0.1 |
| Polychaete | *Paraprionospio pinnata* | 0.5 |  | Polychaete | *Mediomastus californiensis* | 0.1 |
| Polychaete | *Magelona roijai* | 0.5 |  | Polychaete | Capitellidae | 0.1 |
| Crustacean | *Cyclaspis sp* | 0.5 |  | Nemertea | Nemertea | 0.1 |
| Polychaete | Orbiniidae | 0.5 |  | Polychaete | *Glycinde solitaria* | 0.1 |
| Polychaete | *Leitoscoloplos fragilis* | 0.5 |  | Polychaete | *Sthenelais limicola* | 0.1 |
| Amphipod | *Monocorophium sp* | 0.4 |  | Polychaete | *Goniada littorea* | 0.1 |
| Bivalve | *Anandara transversa* | 0.4 |  | Bivalve | *Ameritella versicolor* | 0.1 |
| Crustacean | Shrimp | 0.3 |  | Polychaete | Cirratulidae | 0.1 |
| Bivalve | *Mulinia lateralis* | 0.3 |  | Gastropod | *Nassarius acutus* | 0.1 |
| Bivalve | *Ameritella versicolor* | 0.3 |  | Hemichordata | Hemichordate | 0.1 |
| Polychaete | *Aricidea sp.* | 0.3 |  | Cephalochordate | *Branchiostoma floridae* | 0.1 |
| Polychaete | Spionidae | 0.3 |  | Crustacean | *Ampelisca sp* | 0.1 |
| Polychaete | *Paramphinome* sp. B | 0.3 |  | Cnidaria | *Paranthus rapiformis* | 0.1 |
| Amphipod | *Amphipod* B | 0.2 |  | Crustacean | *Pinnixa sp* | <0.1 |
| Polychaete | *Gyptis vittata* | 0.2 |  | Polychaete | *Aricidea sp* | <0.1 |
| Gastropod | *Nassarius acutus* | 0.2 |  | Crustacean | *Monocorophium sp* | <0.1 |
| Crustacean | *Albunea paretii* | 0.2 |  | Amphipod | *Amphipod B* | <0.1 |
| Crustacean | Hermit crab | 0.1 |  | Echinoderm | *Amphipholis squamata* | <0.1 |
| Gastropod | *Euspira pallida* | 0.1 |  | Cnidaria | Sea anemone | <0.1 |
| Polychaete | *Scoloplos sp* | 0.1 |  | Crustacean | Crab (unknown juv.) | <0.1 |
| Polychaete | Eulepethidae | 0.1 |  | Polychaete | Sigalionidae | <0.1 |
| Polychaete | *Polydora ligni* | 0.1 |  | Polychaete | *Mediomastus* sp | <0.1 |
| Polychaete | *Scoloplos* sp. B | 0.1 |  | Polychaete | *Scoloplos rubra* | <0.1 |
| Polychaete | *Sigambra tentaculata* | 0.1 |  | Gastropod | *Euspira pallida* | <0.1 |
| Polychaete | Cirratulidae | 0.1 |  | Bivalve | *Dosinia discus* | <0.1 |
| Crustacean | Copepod | 0.1 |  | Crustacean | *Callinectes similis* | <0.1 |
| Crustacean | *Ovalipes floridanus* | 0.1 |  | Polychaete | *Cirrophorus lyra* | <0.1 |
| Crustacean | Unknown Crab | 0.1 |  | Nematoda | Nematode | <0.1 |
| Polychaete | *Sigalion spinosus* | 0.1 |  | Polychaete | *Aglaophamus verrilli* | <0.1 |
| Polychaete | *Aglaophamus verrilli* | 0.1 |  | Polychaete | Onuphidae | <0.1 |
| Polychaete | Onuphidae | 0.1 |  | Polychaete | *Grubeulepis mexicana* | <0.1 |
| Polychaete | *Grubeulepis mexicana* | 0.1 |  | Crustacean | *Ovalipes floridanus* | <0.1 |
| Platyhelminth | Platyhelminthes | 0.1 |  | Polychaete | Ampharetidae | <0.1 |
| Polychaete | Sigalionidae | 0.1 |  | Polychaete | *Tharyx annulosus* | <0.1 |
| Bivalve | *Solen viridis* | <0.1 |  | Crustacean | Cumacea | <0.1 |
| Bivalve | *Strigilla pisiformis* | <0.1 |  | Polychaete | *Travisia hobsonae* | <0.1 |
| Bivalve | Lucinidae | <0.1 |  | Polychaete | Orbiniidae | <0.1 |
| Bivalve | Veneridae | <0.1 |  | Polychaete | *Apoprionospio dayi* | <0.1 |
| Crustacean | *Pinnixa sp* | <0.1 |  | Polychaete | *Leitoscoloplos fragilis* | <0.1 |
| Cnidaria | *Paranthus rapiformis* | <0.1 |  | Polychaete | *Mesochaetopterus capensis* | <0.1 |
| Bivalve | *Dosinia discus* | <0.1 |  | Gastropod | Gastropod | <0.1 |
| Polychaete | *Loimia medusa* | <0.1 |  | Crustacean | Hermit crab | <0.1 |
| Polychaete | *Magelona sp.* | <0.1 |  | Polychaete | *Lepidasthenia sp* | <0.1 |
| Gastropod | *Oliva sayana* | <0.1 |  | Platyhelminth | *Platyhelminthes* | <0.1 |
| Gastropod | *Neverita duplicata* | <0.1 |  | Gastropod | *Neverita duplicata* | <0.1 |
| Crustacean | *Callinectes similis* | <0.1 |  | Polychaete | *Phyllodoce groenlandica* | <0.1 |
| Polychaete | *Phyllodoce groenlandica* | <0.1 |  | Bivalve | *Strigilla pisiformis* | <0.1 |
| Polychaete | Ophellidae | <0.1 |  | Annelid | Sipuncula | <0.1 |
| Polychaete | *Arenicola sp.* | <0.1 |  | Polychaete | *Glycera americana* | <0.1 |
| Polychaete | *Magelona* sp B | <0.1 |  |  |  |  |
| Polychaete | *Owenia fusiforms* | <0.1 |  |  |  |  |
| Polychaete | *Polyodontes sp* | <0.1 |  |  |  |  |
| Polychaete | [*Prionospio pygmaeus*](https://www.marinespecies.org/aphia.php?p=taxdetails&id=338539) | <0.1 |  |  |  |  |
| Polychaete | *Spiochaetopterus costarum* | <0.1 |  |  |  |  |
| Gastropod | *Zebina browniana* | <0.1 |  |  |  |  |
